# The Interplay Between Coronavirus and Type I IFN Response

**DOI:** 10.3389/fmicb.2021.805472

**Published:** 2022-03-04

**Authors:** Wenxiang Xue, Chan Ding, Kun Qian, Ying Liao

**Affiliations:** ^1^Department of Avian Diseases, Shanghai Veterinary Research Institute, Chinese Academy of Agricultural Sciences, Shanghai, China; ^2^Jiangsu Co-innovation Center for Prevention and Control of Important Animal Infectious Diseases and Zoonoses, Yangzhou University, Yangzhou, China

**Keywords:** innate immunity, coronavirus, type I IFN response, immune evasion, viral replication

## Abstract

In the past few decades, newly evolved coronaviruses have posed a global threat to public health and animal breeding. To control and prevent the coronavirus-related diseases, understanding the interaction of the coronavirus and the host immune system is the top priority. Coronaviruses have evolved multiple mechanisms to evade or antagonize the host immune response to ensure their replication. As the first line and main component of innate immune response, type I IFN response is able to restrict virus in the initial infection stage; it is thus not surprising that the primary aim of the virus is to evade or antagonize the IFN response. Gaining a profound understanding of the interaction between coronaviruses and type I IFN response will shed light on vaccine development and therapeutics. In this review, we provide an update on the current knowledge on strategies employed by coronaviruses to evade type I IFN response.

## Introduction

Coronavirus disease 2019 (COVID-19) caused by severe acute respiratory syndrome coronavirus 2 (SARS-CoV-2) has been spreading worldwide since December 2019 and has led to the ongoing pandemic, which has infected 271 million individuals and has resulted in 5.32 million deaths until December 14, 2021. Coronaviruses also pose a fatal threat to animal health, including livestock, poultry, and pets, causing enormous economic losses. Given the high similarity between SARS-CoV-2 and bat SARS-CoV-like coronaviruses, it is not surprising that a few mutations in the S protein may confer animal coronaviruses the ability to jump from animals to humans (Andersen et al., 2020; Malaiyan et al., 2021). The innate immune responses are the host’s first defense line and are essential for controlling and eliminating virus infection. It is essential to understand the mechanisms employed by coronaviruses to evade or antagonize the innate immune system. To prevent and control coronaviruses-related diseases, we summarize the recent research on human and animal coronaviruses’ innate immune evasion strategies.

### Knowledge Card of the Coronavirus

Coronaviruses belong to the family *Coronaviridae* and are divided into four genera. α*-coronavirus* and β*-coronavirus* only infect mammals, while γ*-coronavirus* and δ*-coronavirus* (but not all) are avian coronaviruses (Gorbalenya et al., 2004; Woo et al., 2010, 2012). The three highly pathogenic human coronaviruses SARS-CoV, SARS-CoV-2, and Middle East respiratory syndrome (MERS-CoV) belong to β*-coronavirus* and cause severe symptoms in the lung, with high mortality. The low or mild pathogenic human coronaviruses such as HCoV-OC43, HCoV-HKU1, HCoV-229E, and HCoV-NL63 continually circulate in the human population worldwide and produce mild symptoms of the common cold (Fouchier et al., 2004; Van der Hoek et al., 2004, 2006; Woo et al., 2005).

Coronaviruses also impose great threat to economic animals, resulting in tremendous economic losses (Cui et al., 2019; Adachi et al., 2020). For example, the infectious bronchitis virus (IBV) has been circulating in poultry farms for many years since the 1930s, with high mortality, and is highly contagious. It mainly causes a decline in laying eggs and meat production, resulting in a substantial economic loss in the poultry industry (Cavanagh, 2007; Jordan, 2017). Coronaviruses also affect porcine breeding and cattle raising. Porcine epidemic diarrhea virus (PEDV) has emerged recently and causes diarrhea in pigs, with 100% mortality in piglets (Wood, 1977; Jung et al., 2020). Transmissible gastroenteritis virus (TGEV) is another coronavirus which has circulated in porcine farm for many years and causes diarrhea in young pigs (Katsuda et al., 2006; Xia et al., 2018). In the cattle farm, bovine coronavirus (BCoV) is responsible for severe enteritis in young calves (Saif, 2010). Coronaviruses are also present in domestic pets such as cats, dogs, and ferrets, among which, feline infectious peritonitis coronavirus (FIPV) causes high mortality in felines (Tekes and Thiel, 2016).

Coronaviruses have the largest genomes of all known RNA viruses, ranging from 25 to 32 kb (Mihindukulasuriya et al., 2008). This virus family contains a positive-sense, single-stranded, linear genome that possesses a conserved gene order with 5′-replicase-Spike(S)-Envelope(E)-Membrane(M)-Nucleocapsid(N)-3′. Viral replicase gene covers about two-thirds of 5′ genome, with open reading frames (ORFs) 1a and 1b. Replicase gene produces polyprotein 1a and 1ab, which are involved in coronavirus replication after cleaving into 15–16 nonstructural proteins (nsps) (Wang et al., 2020). Papain-like protease (PLpro, nsp3) and 3C-like protease (3CLpro, nsp5) are responsible for cleaving the polyprotein 1a and 1ab. Nsps are conserved across coronaviruses, except for nsp1, encoded only in α*-coronavirus* and β*-coronavirus*, which is associated with suppressing host protein translation (Nakagawa and Makino, 2021). IBV nsp2 is a weak protein kinase R (PKR) inhibitor, involving in evading host innate immune system (Wang et al., 2009). Double-membrane vesicles (DMVs) are the “replication factory” of coronaviruses, closely associated with nsp3 (PLpro), nsp4, and nsp6 (Thiel et al., 2003; Neuman et al., 2014; Neuman, 2016; Oudshoorn et al., 2017). Moreover, coronavirus replicase gene encodes RNA-dependent RNA polymerase (RdRp, nsp12), helicase (nsp13), and three RNA-processing enzymes 3′-5′ exonuclease (nsp14), poly(U)-specific endonuclease (nsp15) and 2′-O-methyltransferase (nsp16) (Decroly et al., 2008; Chen et al., 2009; Zust et al., 2011; Kindler and Thiel, 2014; Fehr and Perlman, 2015).

**FIGURE 1:**
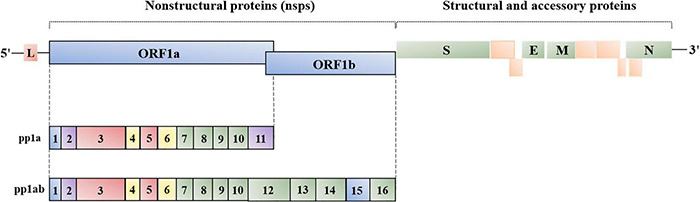
Genome organization of coronavirus. L, leader; ORF1a/1b, replicase, encoding nsp1-16 (γ- and δ*-coronavirus* lack nsp1); S, spike; E, envelope; M, membrane; N, nucleocapsid. The location and number of accessory proteins vary in different coronaviruses.

The downstream of the replicase gene is the last one-third genome encoding structural and accessory proteins. These proteins are not directly translated from genomic RNA (gRNA), but from 3′ nested sub-genomic mRNAs (sgRNAs), which are generated by dis-continuous transcription (Masters, 2006; Hidalgo et al., 2021). Genes encoding accessory protein are interspersed within genes encoding structural protein. Different genera of coronaviruses harbor different numbers of accessory protein genes, and the accessory proteins are not necessary for coronavirus replication (Wang et al., 2020). Structural proteins (S, M, E, and N) are mainly involved in the initial (attachment and entry) and last (assembly and release) steps of the coronavirus life cycle. The start signal of the coronavirus’ life cycle is the binding to specific cell surface receptors by the S protein. It is the most diverse protein among coronavirus structural proteins and is responsible for cross-species transmission when mutation happens. This is, a type I membrane glycoprotein with two functional subunits, S1 and S2, which are in charge of receptor binding and membrane fusion, respectively (Yurkovetskiy et al., 2020). For SARS-CoV and SARS-CoV-2, the cell surface receptor is angiotensin converting enzyme 2 (ACE2) (Mathewson et al., 2008; Benton et al., 2020; Coate et al., 2020), whereas, for MERS-CoV, the receptor is dipeptidyl peptidase 4 (DPP4) (Van Doremalen et al., 2014; Lim et al., 2016; Letko et al., 2020). During coronavirus replication, a replication–transcription complex (RTC) is formed by gRNA, N protein, and replicases, to support gRNA replication and mRNA transcription, associated with DMVs (Gosert et al., 2002; Snijder et al., 2006; Knoops et al., 2008; Ulasli et al., 2010; Lundin et al., 2014; Avila-Perez et al., 2016). After all materials are prepared, the M protein triggers virion assembly in the ER-Golgi intermediate compartment (ERGIC) (Klumperman et al., 1994). During the entire assembly process, the M protein acts as a scaffold protein to interact with the S protein and N protein to recruit structural components, gRNA is brought into the virion by N protein; while the M–E protein interaction is responsible for membrane bending (Lim and Liu, 2001; Fung and Liu, 2019). Finally, mature virions are budded from ERGIC are released by exocytosis (Neuman et al., 2011; McBride et al., 2014; Ujike and Taguchi, 2015; Figure 1).

When SARS-CoV and MERS-CoV infect mice, rapid virus replication and delayed type I IFN expression are observed in the lungs. The pretreatment of IFNα/β protects mice from lethal clinical diseases, implying that coronaviruses are sensitive to IFN treatment (Channappanavar et al., 2016, 2019). In addition, the delayed IFN response is also observed during IBV, PEDV, MHV, and PDCoV infection (Roth-Cross et al., 2007; Cao et al., 2015; Kint et al., 2015b; Luo et al., 2016). Thus, inhibiting type I IFN response is conserved among coronaviruses and is essential for the successful infection of coronavirus.

### Type I IFN Response

As a pioneer in eradicating pathogens, type I IFN response plays an important role in the innate immune system. The type I IFN signaling pathway is mainly composed of the IFN induction pathway and the Janus kinase/signal transducer and activator of transcription (JAK/STAT) pathway; the latter is responsible for the expression of IFN-stimulated genes (ISGs). Activating the type I IFN response is a delicate and complicated process that involves subtle molecular interactions, the formation of functional complexes, the post-translational modifications, and the assistance of the cytoplasmic–nuclear transport system.

#### IFN Induction Pathway

The key to turn on the type I IFN system is through “aberrant” nucleic acids, the pathogen-associated molecular patterns (PAMPs), which are recognized by host pattern recognition receptors (PRRs) (Wu and Chen, 2014). The coronavirus PAMP is double-stranded RNA (dsRNA) produced during virus replication, which is usually detected by cytosolic retinoic acid-inducible gene I (RIG-I)-like receptors (RLRs), including RIG-I or melanoma differentiation-associated protein 5 (MDA5) (Zielecki et al., 2013). After using their helicase and C-terminal regulatory domain to sense dsRNA, RIG-I or MDA5 undergoes a conformational change. RIG-I forms the tetramer *via* N-terminal tandem caspase activation and recruitment domains (2CARD), which are modified by K63-polyubiquitin chains to stabilize the RIG-I 2CARD tetramer. For MDA5, 2CARD forms large oligomers by self-association in a concentration-dependent manner (Zerbe et al., 2020). Subsequently, the similar CARD domain of mitochondrial antiviral signaling protein (MAVS) interacts with 2CARD of RLRs, leading to tripartite motif-containing protein 31 (TRIM31)-mediated K63-linked polyubiquitination of MAVS and then the prion-like aggregation of MAVS CARD, which is essential for the biological function of MAVS (Oshiumi et al., 2010; Hou et al., 2011; Peisley et al., 2014; Liu et al., 2017). Activated MAVS recruits TRAFs, which are pre-associated with TBK1/IKKε; next, TBK1 is activated by MAVS-TRAFs-mediated oligmerization/auto-phosphorylation and NEMO-IKKβ-mediated phosphorylation, resulting in the formation of the TBK1 complex (containing TBK1, IKK i/ε, and NEMO) (Liu S. et al., 2013; Fang et al., 2017). MAVS is phosphorylated by the TBK1 complex, thereby gaining the ability to recruit IRF3. TBK1 promotes IRF3 phosphorylation and homodimerization, and then IRF3 is translocated into the nucleus to induce type I IFN transcription (Liu et al., 2015; Oshiumi, 2020).

#### JAK-STAT Pathway

IFN-α receptor (IFNAR) consists of two subunits IFNAR1 (low affinity) and IFNAR2 (high affinity) (Piehler et al., 2012; Schreiber and Piehler, 2015). IFNα/β binds to IFNAR1 or IFNAR2 to form a binary complex, bringing the receptor-associated kinases JAK1 and TYK2 into proximity, resulting in kinase transphosphorylation and subsequent phosphorylation of tyrosine on IFNAR1 and IFNAR2 (Deonarain et al., 2002). Phospho-tyrosine residues on IFNAR1 and IFNAR2 provide a docking site for STAT proteins (Hertzog and Williams, 2013). Once recruited, STAT proteins are phosphorylated and form homo- or hetero-dimers. The STAT dimers are released from the IFNAR and then associate with IFN regulatory factor 9 (IRF9) to form a complex, ISGF3. ISGF3 is transported into the nucleus and binds to a specific promoter, IFN-stimulated regulatory elements (ISREs), leading to expression of hundreds of ISGs (Heim et al., 1995; Leung et al., 1995; Darnell, 1997; Li et al., 1997; Hebenstreit et al., 2005; Fink and Grandvaux, 2013; McNab et al., 2015).

ISGs are grouped into three functional classes: type I IFN response reinforcement, antiviral factors, and IFN desensitization. ISGs enhance type I IFN response in different ways. PRRs are one type of ISGs responsible for the detection of PAMPs, and IRFs are the type of ISGs promoting target genes’ transcription. There is also a type of ISGs that reinforce the type I IFN response by assisting signal transduction. For example, the upregulation of E3 ubiquitin ligase TRIM21 through the JAK-STAT pathway promotes K27-linked ubiquitination of MAVS, thereby promoting the ability of MAVS to recruit TBK1 (Xue et al., 2018). Most antiviral ISGs fight against the virus by intervening virus life cycle. For example, myxovirus resistance (Mx), cholesterol-25-hydroxylase (CH25H), and IFN-inducible transmembrane (IFITM) protein inhibit the early stage of the virus life cycle, virus entry (Gao et al., 2010; Park and Scott, 2010; Haller and Kochs, 2011; Anafu et al., 2013; Liu S. Y. et al., 2013; Wilkins et al., 2013; Zang et al., 2020). OAS/RNaseL and PKR detect viral RNA to restrict virus replication at the transcriptional and translational levels, respectively (Chakrabarti et al., 2011; Munir and Berg, 2013; Gal-Ben-Ari et al., 2018). In addition, virus budding and release can be restricted by Viperin and Tetherin (Wang et al., 2007; Perez-Caballero et al., 2009; Szretter et al., 2011; Swiecki et al., 2013). The last ISGs group is responsible for IFN desensitization to avoid over- or persistent activation of the JAK-STAT pathway. SOCS protein is known as a JAK-STAT pathway inhibitor. SOCS1 associates with the IFNAR to prevent STATs from attaching to the receptor, while SOCS3 binds JAKs to inhibit the activity of these kinases (Hong and Carmichael, 2013). Unlike SOCS proteins, USP18 binds IFNAR2 to block the activation of the JAK-STAT pathway induced by IFNα (Francois-Newton et al., 2011). In addition, the protein inhibitor of activated STAT1 (PIAS1) is a broad inhibitor of the type I IFN system. Many studies have shown that PIAS1 inhibits the DNA binding activity of transcription factors, including STAT1, IRF3, and p65 (Liao et al., 2000; Liu et al., 2005; Shuai and Liu, 2005; Shuai, 2006; Li et al., 2013).

#### Nucleocytoplasmic Transport System Involved in Type I IFN Response

To perform the function as a transcription factors, the cytoplasmic STATs and IRF3 must gain access into the nucleus to bind specific DNA promoters. The nuclear translocation process is closely related to the nucleocytoplasmic transport system (Springhower et al., 2020). Transport direction through the nuclear pore complex (NPC) depends on the signal sequence on the cargo proteins: the nuclear localization signal (NLS) guides cargos into the nucleus and the nuclear export signal (NES) guides cargos into the cytoplasm. Karyopherin subunit α (KPNA) acts as an adaptor protein that binds NLS of cargo proteins, and then the KPNA–cargo complex combines with Karyopherin subunit β1 (KPNB1) to form the KPNA:KPNB1:Cargo complex. KPNB1 interacts with NPC and eventually transports cargos into the nucleus (Chook and Blobel, 2001; Cautain et al., 2015). In general, KPNA2, KPNA3, and KPNA4 mediate the import of phospho-IRF3 into the nucleus (Reich, 2002; Cai et al., 2020), whereas the ISGF3 complex enters the nucleus *via* KPNA1 (Sekimoto et al., 1997; Nardozzi et al., 2010). After transcription factors have completed their mission, they must exit from the nucleus. Most transcription factors are exported into the cytoplasm through *exportin1* (CRM1), with the widest range of known substrates (Fornerod et al., 1997; Kutay and Guttinger, 2005). When phospho-STAT1 is dephosphorylated by nuclear phosphatase TC45, the NES of STAT1 binds to CRM1 and goes through the NPC to the cytoplasm (McBride et al., 2000; Ten Hoeve et al., 2002). Another interesting example is the nucleocytoplasmic shuttling of the STAT2-IRF9 complex. IRF9 contains a bipartite basic NLS that directs the unphosphorylated STAT2-IRF9 (U-STAT2-IRF9) complex into the nucleus, while STAT2 harbors a functional and dominant NES that guides the return of the U-STAT2-IRF9 complex to the cytoplasm. As a result, the U-STAT2-IRF9 complex is normally located in the cytoplasm (Lau et al., 2000; Banninger and Reich, 2004).

### IFN Evasion Strategies of Coronavirus

**FIGURE 2:**
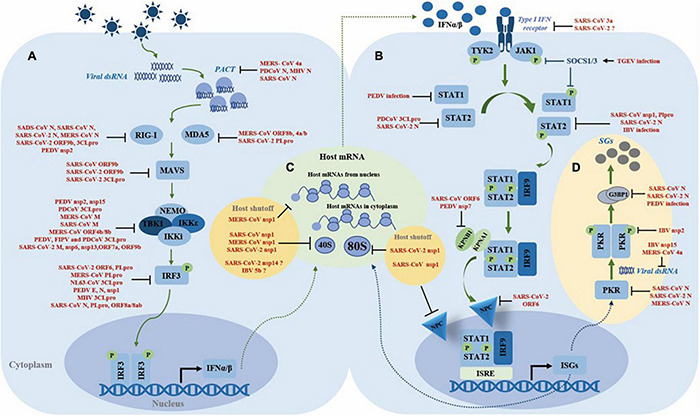
Summary of the mechanisms of coronaviruses antagonizing type I IFN system. **(A)** Inhibition of IFN induction. **(B)** Inhibition of the JAK-STAT pathway. **(C)** Manipulation of the host translation machinery. **(D)** Regulation of stress granules formation.

To guarantee their successful replication in host cells, coronaviruses have evolved rigorous and precise strategies to evade the host’s innate immune system, especially type I IFN response. Viral proteins are the main executors of immune evasion strategies during the infection process. For instance, among the viral proteins encoded by SARS-CoV or SARS-CoV-2, at least 8 proteins have been confirmed harboring the activity of hindering type I IFN response (Totura and Baric, 2012; Lei et al., 2020). Here, we will summarize the strategies exploited by the coronaviruses to evade type I IFN response (Figure 2).

### Inhibition of IFN Induction

Inhibiting IFNα/β production is the first and essential step for coronavirus to evade the host immune defense. Due to the delayed type I IFN response, the host misses the best timing to clear the virus. In turn, the virus achieves successful replication (Rose et al., 2010; Menachery et al., 2014; Kint et al., 2015b). Escaping viral RNA recognition, inhibiting innate immune sensors, targeting the TBK1 complex, and interfering with IRF3 are the main strategies used by coronaviruses to inhibit IFNα/β expression.

#### Escaping Viral RNA Recognition

During replication, viral RNA or intermediate products like dsRNA are recognized by host immune sensors, such as RIG-I, MDA5, or TLR. Coronaviruses have evolved three tricks to circumvent host recognition to evade immune sensors’ detection (Ulasli et al., 2010). The first way is “hiding.” Coronavirus creates “a replication factory” called DMVs derived from ER membranes, to hide the viral RNA and to enwrap the replication complex. Nsp3 and nsp4 are involved in the formation of DMVs (Gosert et al., 2002; Knoops et al., 2008; Oudshoorn et al., 2017). DMVs keep viral replication machinery, intermediates, and products away from host sensors, creating an isolated and safe environment for viral replication (Snijder et al., 2006). The second trick is “disguising”. 2′-O-methylation of mRNA is an important modification that provides a molecular marker for host cells to distinguish self mRNA from non-self mRNA, and coronaviruses cleverly evade this discrimination. Coronaviruses have evolved nsp16 with 2′-O-methyltransferase activity, which autonomously modifies viral mRNAs with a 5′ cap structure, mimicking host mRNA. Relevant studies showed that human and mouse coronaviruses without nsp16 2′-O-methyltransferase activity trigger higher expression of type I IFN. Therefore, nsp16 helps the virus to evade the IFN response by modifying viral RNA with a 5′ cap (Zust et al., 2011). The third strategy is “removing”. It has been reported that the highly conserved endoribonuclease nsp15 is responsible for reducing the accumulation of negative-stranded RNA and dsRNA. Recombinant coronavirus lacking the nsp15 endoribonuclease activity produces more dsRNA during infection (Kindler et al., 2017; Gao et al., 2021). It is speculated that coronavirus utilizes its endoribonuclease to cleave its negative-stranded RNA to maintain the ratio of negative-positive stranded RNA to facilitate virus genome replication and transcription, thereby reducing the accumulation of dsRNA and avoiding the activation of host RNA sensors (Deng et al., 2017). This hypothesis was confirmed by Hackbart et al. (2020) who demonstrates that nsp15 cleaves viral 5′-poly(U) negative-stranded RNA at uridine residues to limit the abundance and length of the negative-sense RNA, thereby escaping host immune sensor detection. In conclusion, the DMV, nsp16 and nsp15 play an important role in subverting the recognition of viral RNA by host sensors.

#### Inhibiting the Double-Stranded RNA Sensors RIG-I and Melanoma Differentiation-Associated Protein 5

In addition, coronaviruses also have evolved a series of strategies to directly antagonize the host sensors. For MERS-CoV, co-expression of ORF8b with ORF4a/b significantly downregulates the protein level of MDA5 (Lee et al., 2019), whereas for SARS-CoV-2, current studies show that papain-like protease (PLpro) antagonizes ISG15-dependent MDA5 activation through de-ISGylation (Liu et al., 2021a). Several reports show that the N protein from SARS-CoV, SARS-CoV-2, and MERS-CoV interferes with K63-linked ubiquitination of RIG-I by interacting with TRIM25 E3 ubiquitin ligase, thereby preventing the activation of RIG-I; as for SADS-CoV, the N protein directly associates with RIG-I and leads to RIG-I degradation by ubiquitination (Hu et al., 2017; Chang et al., 2020; Chen et al., 2020; Liu et al., 2021b; Oh and Shin, 2021). SARS-CoV-2 main protease 3CLpro not only cleaves the N-terminal amino acids from RIG-I and deprives its ability to activate MAVS, but also promotes the ubiquitination and proteasome-mediated degradation of MAVS; in addition, the SARS-CoV-2 unique accessory protein 9b interferes with RIG-I and MAVS interaction (Kouwaki et al., 2021; Liu et al., 2021c). PACT is a dsRNA-binding protein that binds dsRNA to activate RIG-I or MDA5, thereby initiating the type I IFN induction pathway. The N protein from PDCoV, MHV, and SARS-CoV directly interacts with PACT, thus impeding the association of RIG-I or MDA5 with dsRNA or/and PACT (Ding et al., 2017; Chen J. et al., 2019), whereas the MERS-CoV 4a protein contains a dsRNA-binding domain that competes with PACT for dsRNA binding (Siu et al., 2014b). The E3 ubiquitin ligase FBXW7 is verified as an innate antiviral factor capable of enhancing the expression of RIG-I and TBK1. A recent study reports that PEDV nsp2 interacts with and targets FBXW7 for degradation through the K48-linked ubiquitination, thereby reducing the levels of RIG-I and TBK1, which are confirmed *in vivo* (Li et al., 2021; Figure 2A). Above reports show that the N protein’s antagonism to the dsRNA sensors is conserved in several coronaviruses. As the N protein is a structural and functional conserved protein, we speculate that the N protein plays an important role in innate immune evasion during different genera coronaviruses infection, which needs further investigation. The virus proteases, nsps, and unique accessory proteins play a certain role in the antagonism of dsRNA sensors.

#### Targeting the TBK1 Complex

Once immune sensors have been activated, the next step is the formation of the TBK1 complex (containing TBK1, IKK i/ε, and NEMO). Coronaviruses also use their proteins as weapons to prevent TBK1 complex formation. For example, SARS-CoV-2 M, nsp6, and nsp13 directly interact with TBK1 to suppress IRF3 phosphorylation (Xia et al., 2020; Zheng et al., 2020); SARS-CoV-2 unique accessory protein ORF7a destabilizes TBK1; while PEDV endoribonuclease nsp15 targets TBK1 mRNA for degradation (Wu et al., 2020; Kouwaki et al., 2021). SARS-CoV and SARS-CoV-2 ORF9b not only inhibit IFN induction pathway by antagonizing MAVS, but also restrain K63-linked ubiquitination of NEMO (Shi et al., 2014; Kouwaki et al., 2021; Wu et al., 2021). MERS-CoV M protein destroys the interaction of TRAF3, TBK1, and IKKε, and so do SARS-CoV M and PDCoV 3CLpro (Siu et al., 2009, 2014a; Lui et al., 2016). Unlike MERS-CoV ORF4b, which binds TBK1 and IKKε directly (Yang et al., 2015), MERS-CoV ORF8b competes with IKKε for interacting with HSP70 that is vital for activation of IKKε, thus blocking the downstream signal transduction (Wong et al., 2020). PEDV, FIPV, and PDCoV 3CLpro cleaves NEMO at glutamine 231 (Q231), thereby inhibiting TBK1 complex formation (Wang et al., 2016; Zhu et al., 2017a; Chen S. et al., 2019; Figure 2A). Altoghther, the M protein, main protease 3CLpro, accessory proteins, and several nsps play the major role in antagonizing the TBK1-NEMO-IKKε complex, by impeding protein–protein interaction, preventing the post-translation modification, such as ubiquitination, or directly cleaving the signaling molecules.

#### Interfering With IFN Regulatory Factor 3

Inhibiting nuclear translocation of IRF3 is a common trick for the coronaviruses to compromise type I IFN expression. PLpro from MERS-CoV, SARS-CoV, and HCoV-NL63 not only harbor the proteolytic processing but also process deubiquitinating activity, which have been confirmed to affect K48- and K63-linked ubiquitination and ISG15-linked ISGylation of host proteins, thereby inhibiting type I IFN signaling. Furthermore, these PLpro impair IRF3 activation and its nuclear translocation, independently of its protease activity (Barretto et al., 2005; Chen et al., 2007; Yang et al., 2014). SARS-CoV-2 PLpro directly cleaves IRF3 to prevent the transcription of type I IFN (Moustaqil et al., 2021). SARS-CoV ORF8a/8ab protein mediate ubiquitin-dependent rapid degradation of IRF3, whereas PEDV and SARS-CoV-2 N protein curb IRF3 phosphorylation and nuclear translocation by sequestering the interaction between TBK1 and IRF3 interaction (Ding et al., 2014; Wong et al., 2018; Zhao et al., 2021). PEDV E protein is also associated with IRF3 directly to inhibit IRF3 nuclear translocation (Zheng L. et al., 2021). The MHV PLpro targets TBK1 by deubiquitination, thereby reducing its kinase activity and forming an inactive complex with TBK1 and IRF3 in the hypo-phosphorylated form in the cytoplasm (Wang et al., 2011). SARS-CoV-2 ORF6 chooses another way to inhibit nuclear translocation of IRF3, by binding importin Karyopherin α2 (KPNA2), a nuclear transport importer, to antagonize the expression of type I IFN (Xia et al., 2020). Even if phospho-IRF3 successfully enters the nucleus, PEDV nsp1 induces degradation of CREB-binding protein (CBP) through ubiquitination, thereby disrupting the binding of IRF3 to CPB and ultimately inhibiting type I IFN expression (Zhang et al., 2016; Figure 2A). It seems that the PLpro and N protein play the major role in impeding the IRF3 activation or nuclear translocation.

In summary, coronaviruses employ several conserved strategies to evade host sensor’s detection of viral RNA or dsRNA, by forming DMVs (nsp3 and nsp4 are involved), by disguising viral RNA (nsp16 is involved), or by removing dsRNA/negative intermediates (nsp15 is involved). Several conserved viral proteins are involved in antagonism of innate immune responses: the N protein employs similar strategies to interfere with the activation of sensors; protease PLpro and 3CLpro are involved in many steps of type I IFN signaling, by directly the signaling proteins or by promoting the degradation of signaling proteins via manipulation of the ubiquination modification. These conserved viral proteins are excellent targets for therapeutics and vaccine design. The compounds targeting nsp15 (Canal et al., 2021; Kumar et al., 2021; Sixto-Lopez and Martinez-Archundia, 2021), nsp16 (Krafcikova et al., 2020; Lin et al., 2020; Rosas-Lemus et al., 2020), PLpro (Baez-Santos et al., 2015; Jo et al., 2020; Paul et al., 2021), and 3CLpro (Hakami et al., 2021; Milligan et al., 2021) are under screening and investigation.

### Inhibition of the JAK-STAT Pathway

Even though IFNα/β are successfully expressed, the coronavirus still employs strategies to impede the JAK-STAT pathway (Figure 2B). SARS-CoV accessory protein 3a induces serine phosphorylation of IFNAR1, resulting in ubiquitination and lysosomal degradation of IFNAR1 (Minakshi et al., 2009). IFNAR1 ubiquitination and degradation can also be induced by SARS-CoV-2 infection, but the precise mechanism is unknown yet (Chen et al., 2021). During TGEV infection, the level of miR-30a-5p is downregulated and then the negative regulators SOCS1/3 are increased to block the type I IFN response (Ma et al., 2018). Coronaviruses also employ multiple ways to inhibit STAT1 and STAT2 nuclear translocation, including downregulating the molecules involved, interfering with post-translational modifications of signaling molecules, and disrupting the function of nuclear transport receptors. PEDV infection causes degradation of STAT1 (Guo et al., 2016), while PDCoV 3CLpro cleaves STAT2 *via* its protease activity (Zhu et al., 2017b). SARS-CoV-2 N interacts with and downregulates STAT2 protein through an unknown mechanism (Chen et al., 2021). SARS-CoV nsp1 inhibits STAT1 phosphorylation and nuclear translocation, and IBV infection STAT1 phosphorylation and nuclear translocation by unclear mechanisms (Wathelet et al., 2007; Kint et al., 2015a). Since γ*-coronavirus* lacks nsp1, IBV definitely encodes an alternative protein to play a similar role to nsp1 (Kint et al., 2015a). STAT1 phosphorylation is also impeded by SARS-CoV PLpro, which disrupts the interaction between extracellular signal-regulated kinase (ERK) 1 and STAT1 (Li et al., 2011). Both SARS-CoV and SARS-CoV-2 ORF6 impact the nuclear transport system in diverse ways: SARS-CoV ORF6 interacts with KPNA2 and promotes KPNA2 to hijack KPNB1 to the ER/Golgi membrane, resulting in the shortage of KPNB1 that is responsible for carrying the ISGF3 complex to the nucleus (Frieman et al., 2007); while SARS-CoV-2 ORF6 inhibits STAT1 and STAT2 nuclear translocation by preventing the ISGF3/KPNA1/KPNB1 complex from docking with the Nup98-Rae1 nuclear pore complex (Miorin et al., 2020). Another study showed that PEDV nsp7 disrupts the interaction between KPNA1 and STAT1/2, hence impeding ISGF3 nuclear translocation (Li et al., 2020). Interestingly, recent studies showed that in contrast to the decrease of STAT1/2 phosphorylation, high concentration of SARS-CoV-2 N increases phosphorylation by low concentration of SRAS-CoV-2 N protein and nuclear translocation of STAT1/2 (Mu et al., 2020; Zhao et al., 2021).

Although coronaviruses use a series of strategies to inhibit ISGs expression, the JAK-STAT pathway is activated during cytokine release syndrome (CRS) caused by SARS-CoV and SARS-CoV-2 (Schwartz et al., 2017; Huang et al., 2020; Zhou et al., 2020). An animal test showed that lethal doses of SARS-CoV-infected mice lacking IFNα/β receptor displayed a high survival rate and mild clinical disease (Channappanavar et al., 2016). Thus, the activation of the JAK-STAT pathway might be associated with the pathogenicity of coronaviruses. Compared with the number of studies about evasion or antagonism of type I IFN induction by coronaviruses, reports on the antagonism of the JAK-STAT pathway are limited. One reason for this phenomenon is the lower cost-effectiveness, since the JAK-STAT pathway is generally silent during coronavirus infection due to the failure of IFNα/β induction. Another reason is the research on the JAK-STAT pathway is a contradictory and tough task. Anyway, the interplay between coronaviruses and the JAK-STAT pathway remains to be further explored.

### Manipulation of the Host Translation Machinery

Shutting down host translation kills two birds with one stone for the coronavirus, by hijacking the host translation system for their own use and inhibiting host antiviral genes expression (Lokugamage et al., 2012, 2015; Tanaka et al., 2012; Kint et al., 2016; Finkel et al., 2021). Inhibiting host antiviral genes expression is an important strategy to antagonize the IFN response. Nsp1 encoded by α- and β*-coronavirus* have similar biological functions to trigger host translation shutoff (Shen et al., 2019). SARS-CoV nsp1 inactivates translation by tightly binding with the 40S ribosomal subunit, while SARS-CoV-2 nsp1 associates with both 40S and 80S ribosomal subunits to achieve host translation shutoff (Kamitani et al., 2009; Schubert et al., 2020; Thoms et al., 2020). In addition, when combining with 40S ribosomes, SARS-CoV nsp1 is also adept at inducing endo-nucleolytic cleavage of 5′ capped non-viral mRNA and triggers the degradation of the cleaved host mRNA by exonuclease Xrn I (Kamitani et al., 2009; Huang et al., 2011; Gaglia et al., 2012). Similar to SARS-CoV nsp1, MERS-CoV nsp1 exerts a parallel mechanism to induce host mRNA degradation (Lokugamage et al., 2015). For SARS-CoV-2 nsp1, which has a high amino acid sequence identity with SARS-CoV nsp1, whether to employ the same way to degrade non-viral RNA remains to be further investigated (Lei et al., 2020; Tidu et al., 2020; Shen et al., 2021). In addition to these common strategies, SARS-CoV nsp1 plays other tricks to prevent RNA nuclear export, by interfering with nucleocytoplasmic transport of RNA binding protein, nucleolin, or by altering the localization of Nup93 (Gomez et al., 2019). SARS-CoV-2 nsp1 directly interacts with mRNA export receptor heterodimer NXF1-NXT1, leading to nuclear retention of host mRNA, further supporting host translation shutdown (Zhang et al., 2021). MERS-CoV nsp1 also inhibits host translation in its unique way, that is, by halting translation of host mRNA through targeting mRNA derived from the nucleus, thereby hijacking the translation machine for the translation of viral RNA that is synthesized in the cytoplasm; however, the detailed mechanism on how MERS-CoV nsp1 specifically recognizes host mRNA transported from the nucleus is unknown (Lokugamage et al., 2015; Figure 2C). As a vital pathogenic determinant, nsp1 is also an excellent drug and vaccine design target. A study demonstrated that SARS-CoV nsp1 with two amino acid residues (KS) deletion possesses a decreased antagonism ability of type I IFN response (Jauregui et al., 2013). Such property may exist in both α- and β*-coronavirus* (Benedetti et al., 2020). Recently, it has been confirmed that several SARS-CoV-2 proteins function as translation inhibitors. The translation inhibition activity of SARS-CoV-2 nsp14 is more significant than nsp1 (Hsu et al., 2021), and nsp16 suppresses global mRNA splicing and prevents the production of mature host mRNA (Banerjee et al., 2020). Since nsp14 and nsp16 are conserved viral proteins among coronaviruses, the mechanisms of these two proteins as translation inhibitors may be a breakthrough in finding common strategies employed by coronaviruses. IBV, a γ*-coronavirus*, which lacks nsp1, also inhibits host translation. It has been shown that IBV accessory protein 5b is responsible for inducing host translation shutoff (Kint et al., 2016). Further research is needed on how coronaviruses block the host translation systems, especially for γ- and δ*-coronavirus*.

### Regulation of Stress Granules Formation

Coronavirus infection is usually accompanied by a host stress response (Kikkert, 2020). When facing infection stress, host cells instinctively limit protein synthesis to restrict virus replication and minimize the cell damage (McCormick and Khaperskyy, 2017). When the protein translation is attenuated, the cell untranslated host mRNA trigger the formation of stress granules (SGs). SGs are large cytoplasmic foci containing stalled 48S preinitiation complexes (PICs) that consist of untranslated mRNAs, translation initiation factors, small ribosomal subunits, and the RNA-binding proteins (RBPs), such as Ras-GAP SH3 domain-binding protein (G3BP) and poly A-binding protein (PABP) (Kedersha et al., 1999; Moser and Fritzler, 2010; Cao et al., 2020). SGs serve as an exhibition of robust translation inhibition and act as a platform for innate immune sensors like PKR, MDA5, and RIG-I, to transmit signaling downstream (Kim et al., 2019; Hofmann et al., 2021). Thus, SGs are also regarded as an antiviral platform involved in PAMP recognition, signaling transduction, and IFN induction (Onomoto et al., 2014; Yoneyama et al., 2016; McCormick and Khaperskyy, 2017; Eiermann et al., 2020; Gao et al., 2021). Since host translation shutoff is the main cause of SG formation, and translation machinery is essential for viral protein synthesis, the coronavirus employs multiple strategies to fine-tune the protein translation efficiency and to manipulate the formation of SGs (Figure 2D).

Most studies have focused on how coronaviruses regulate the PKR-eIF2α-G3BP1 axis, the main pathway responsible for triggering SG formation. Blocking PKR recognition of viral dsRNA and disrupting the assembly of SGs are common techniques used by coronaviruses to manipulate SG formation. MERS-CoV 4a accessory protein binds viral dsRNA to inhibit the activation of PKR, thereby impeding SG formation. MERS-CoV’s inefficient replication was observed while MERS-CoV 4a lost its RNA binding motif. The reason for this phenomenon is that 4a RNA binding defect results in plenty of RNA binding to and *in trans* activation of PKR, and high level of PKR-mediated eIF2α phosphorylation lead to the protein shutoff and formation of SGs, resulting in inefficient viral protein translation (Rabouw et al., 2016; Nakagawa et al., 2018). Hackbart et al. (2020) showed that coronavirus nsp15 cleaves its 5′-poly(U) negative-sense RNA to evade innate immune responses. This mechanism is also utilized by IBV nsp15 to impede the accumulation of dsRNA, thereby decreasing its dsRNA to evade PKR sensor activation (Gao et al., 2021). Gao et al. (2021) also showed that nsp15 of PEDV, TGEV, SARS-CoV, SARS-CoV-2, and MERS-CoV interferes with the formation of SGs induced by chemical stimulation, probably by targeting to host RNA or other host factors. IBV manipulates the protein translation and probably also impedes SG formation by upregulating the expression of phosphatase subunit GADD34 to restrict eIF2α phosphorylation (Wang et al., 2009). The assembly of SGs can be compromised when G3BP1 is impaired. SARS-CoV and SARS-CoV-2 N protein interact with PKR and G3BP1 directly, while MERS-CoV N only associates with PKR to impede SGs assembly (Cai et al., 2021; Luo et al., 2021; Zheng Z. Q. et al., 2021). PEDV infection triggers the cleavage of G3BP1 by caspase-8, which subverts SG formation and promotes virus replication (Sun et al., 2021). Although some studies have reported that SGs induced by TGEV and MHV may be positively correlated with its replication, these studies do not provide sufficient evidence to prove this conclusion, and further studies are needed (Raaben et al., 2007; Sola et al., 2011). The relationship between coronaviruses and SGs appears complicated and needs further study.

## Conclusion

As the first line of defense against pathogens, the innate immune system inevitably becomes the coronaviruses’ primary target, to ensure virus replication and produce infectious progeny. Most coronavirus infection delay type I IFN response, making the host miss the best time to clear the virus. Because of this, the mechanisms of coronaviruses antagonizing the type I IFN response have been a research hotspot. Coronaviruses have been prevalent in livestock and poultry for many years, since the finding of the first coronavirus, IBV, in the 1930s. This virus family mainly causes economic loss in domestic animal farming and is important in the veterinary field. Human coronaviruses only infect the upper respiratory tract and cause mild respiratory symptoms, with low risk to public health, until the outbreak of SARS-CoV in 2003 and MERS-CoV in 2012. With the disappearance of SARS-CoV and MERS-CoV, people seem to have forgotten the suffering of these coronaviruses brought us. The worldwide epidemic of SARS-CoV-2 brought people’s attention back to this virus family: we have never really defeated the coronavirus. Although the vaccine targeting SARS-CoV-2 has been successfully developed and has a protective effect on existing strains, we still face the risk of immune failure, with the emergence of SARS-CoV-2 mutants. To fight the coronavirus, it is important to find common conservative mechanisms for the coronavirus to antagonize the innate immune system. Furthermore, SARS-CoV, MERS-CoV, and SARS-CoV-2 are typical examples of interspecies transmission of viruses from animal to human. Therefore, besides paying attention to human coronaviruses, we should also pay effort to prevent and control these pathogens in animal. Understanding various or common mechanisms employed by coronaviruses to evade or antagonize the host innate immune system could offer therapeutic targets and strategies for controlling coronavirus-associated diseases. This review offers a panoramic view of the mechanisms of fighting against the host innate immune system by human and animal coronaviruses, especially type I IFN response.

## Author Contributions

WX and YL: conceptualization and writing—original draft. CD, KQ, and YL: funding acquisition. YL: project administration and writing—review and editing. CD and YL: supervision. All authors contributed to the article and approved the submitted version.

## Conflict of Interest

The authors declare that the research was conducted in the absence of any commercial or financial relationships that could be construed as a potential conflict of interest.

## Publisher’s Note

All claims expressed in this article are solely those of the authors and do not necessarily represent those of their affiliated organizations, or those of the publisher, the editors and the reviewers. Any product that may be evaluated in this article, or claim that may be made by its manufacturer, is not guaranteed or endorsed by the publisher.
